# A computed tomography cadaveric study of the radiological anatomy of the patella: the size of the patella correlates with bone bridge between tunnels and R angles are introduced for safe tunnel drilling during MPFL reconstruction

**DOI:** 10.1186/s40634-021-00348-9

**Published:** 2021-04-17

**Authors:** Vasileios Raoulis, Ioannis Tsifountoudis, Apostolos Fyllos, Michael Hantes, Michael-Alexander Malahias, Apostolos Karantanas, Aristeidis Zibis

**Affiliations:** 1 Department of Orthopaedic Surgery, University Hospital of Larissa, Larissa, Greece; 2 Department of Anatomy, University Hospital of Larissa, Larissa, Greece; 3 424 General Military Training Hospital, Thessaloniki, Greece; 4 Complex Joint Reconstruction Center, Hospital for Special Surgery, 535 East 72nd Street, New York, NY 10021 USA; 5 Department of Medical Imaging, University Hospital and Radiology, Medical School University of Crete, Heraklion, Greece

**Keywords:** Double-bundle MPFL, Patella tunnels, Patella instability, Cadaveric, Radiological anatomy, Computed tomography

## Abstract

**Purpose:**

To measure the safe range of angles during tunnel drilling and map ideal patella tunnel placement with the use of preoperative computed tomography (CT) scan and compare results after medial patellofemoral ligament (MPFL) reconstruction using a hardware-free patellar fixation technique with two semi-patellar tunnels between a) a free-hand technique, and b) its modification with the use of an anterior cruciate ligament (ACL) tibia aiming device.

**Methods:**

CT scan was performed on 30 fresh-frozen cadaveric knees a) prior to any intervention and b) after MPFL reconstruction. For MPFL reconstruction, specimens were randomly allocated to 1) Group A, which consisted of knees operated with free-hand, hardware-free patellar fixation technique with two semi-patellar tunnels and 2) Group B, which consisted of knees operated on with a technique modification with the ACL tibia device.

**Patellar measurements:**

L1 was the maximal patellar length. L2 was the minimum possible distance of placement for the upper tunnel from the proximal pole of the patella. The maximum bone bridge between tunnels was calculated as half of L1 minus the L2 distance (L1/2-L2). We also measured R1 and R2 angles at the proximal and distal tunnel that represent safe angles at the entry point during tunnel drilling (without breaching the anterior cortex or articular cartilage).

**Results:**

Preoperatively, mean L1 was 3.45 cm (range 3.05–4.52). Mean L2 was 0.62 cm (range 0.49–0.89). The mean maximum possible bone bridge between tunnels (L1/2-L2) was 1.1 cm (range 0.77–1.58).

R1 was 6.05^0^ (range 4.78–7.44), R2 was 6.64^0^ (range 4.57–9.03), and their difference reached statistical significance (*p* = 0.03). Postoperatively, in group A, in 4 out of 15 patellas, multiple attempts were made during tunnel drilling in order to avoid anterior cortex or cartilage breaching. In group B, all tunnels were correctly drilled with the first attempt. Bone bridge between tunnels was significantly shorter postoperatively (0.93 cm, *p* < 0.01).

**Conclusion:**

Small-size patellae correlate with short maximum bone bridge between tunnels, which makes anatomic, double-bundle, hardware-free patella fixation, with two semi-patellar tunnels MPFL reconstruction challenging. Furthermore, R angles create a narrow window to avoid intraoperative breaching, rendering the use of the ACL tibia device an extremely useful instrument.

**Level of evidence:**

II

## Introduction

Medial patellofemoral ligament (MPFL) anatomy and biomechanical properties have been extensively studied in the last twenty years in order to improve surgical reconstruction technique and clinical results [10, 12–15, 17, 20].

Kang et al. introduced the concept of the two functional bundles of MPFL [10]. The horizontal inferior bundle is the main static soft tissue restraint and the oblique superior bundle serves as a dynamic maintenance of patella stability combined with the vastus medialis muscle [10].

From a biomechanical viewpoint, the double-bundle technique has an angular synergy effect that simulates the broad footprint of the MPFL upon the patella, enabling a greater capacity to resist patellar dislocation at the early knee flexion angles [41]. Moreover, the two-point fixation at the patella results in reduced patellar rotation, whereas greater stability can be achieved during flexion and extension [7]. The single-bundle technique may have a greater risk of postoperative apprehension, as this technique cannot restore the broad patellar footprint [11]. The double-bundle MPFL reconstruction remains popular due to better clinical results and its low rates of failure and complications compared with single-bundle reconstruction [33, 39].

The common ground of anatomical MPFL double-bundle reconstruction techniques is the use of gracilis or semitendinosus as the graft of choice [2, 3, 6, 16, 19, 23, 27–35, 38] and that the distal fixation point should be placed at the patella midline (which corresponds to MPFL native attachment). Placement of this fixation point more distally would lead to greater length changes for the distal graft bundle, most evident at deeper flexion angles [12].

The differences of these surgical techniques concern patella fixation, since femoral fixation with a bio-composite screw at the Schöttle point allows isometric adjustments of the graft, resulting in a good clinical outcome [32–34]. Some of the popular techniques include utilization of implants, such as suture-anchors [16, 27, 28, 30, 31] or interference screws for graft fixation of the patella [16, 27, 30, 34]. Others describe anatomic hardware-free patellar fixation, whereby the graft is passed through 2 transpatellar bone tunnels [2, 3, 6, 23], transosseous sutures [42] or a combination of the above [19, 29, 35, 38]. All of these techniques suggest a bone bridge between tunnels of at least 1 cm.

The purpose of the study was to measure the safe range of angles during tunnel drilling, to map ideal patella tunnel placement with the use of preoperative CT scan and compare results after MPFL reconstruction with postoperative CT scans between a) a free-hand, hardware-free patellar fixation technique with two semi-patellar tunnels and b) its modification with the use of an ACL aiming tibia device. Considering the shape and size of the patella and the technical issues described above, we hypothesized that the available working space (maximal bone bridge between tunnels) does not always correspond to 1 cm and consequently, it might not be applicable to small-sized patellae. A secondary hypothesis was that the use of an anterior cruciate ligament (ACL) aiming tibia device would produce more accurate tunnel drilling. The ACL tibia aiming device could contribute in avoiding not only the intra-operative hazard of anterior cortex and articular cartilage breaching, but also multiple attempts at tunnel drilling, compared to a free-hand, hardware-free patellar fixation technique with two semi-patellar tunnels.

## Materials and methods

Following approval from the Institutional Review Board of the University, a total of 30 fresh-frozen cadaveric knees (15 matched pairs) from 15 fresh frozen human cadavers were studied. They consisted of 10 female and 5 male specimens and their mean age was 64.2 years (range 49–80, SD 9.7). There was no medical history of any bone or soft tissue injury, surgery or osteoporosis in any of the specimens. They were obtained through an anatomy donation program and were stored at -21^0^C. CT scan was performed on all specimens a) prior to any intervention (with ideal tunnel mapping) and b) after MPFL reconstruction.

The specimens were randomly allocated to 2 groups, so that knees from the same cadaver were operated with a different technique. Group A consisted of knees operated with free-hand, hardware-free, patellar fixation technique with two transverse semi-patellar tunnels by the same surgeon. Group B consisted of knees operated with the same technique, with the addition of an ACL aiming tibia device for tunnel drilling (instead of free-hand aiming) by a different surgeon. Surgeons were blinded to each other in terms of the purpose of the study and the existence of another group utilizing a different surgical technique.

### Preoperative tunnel mapping (Figs.1 and 2)

**Fig. 1:**
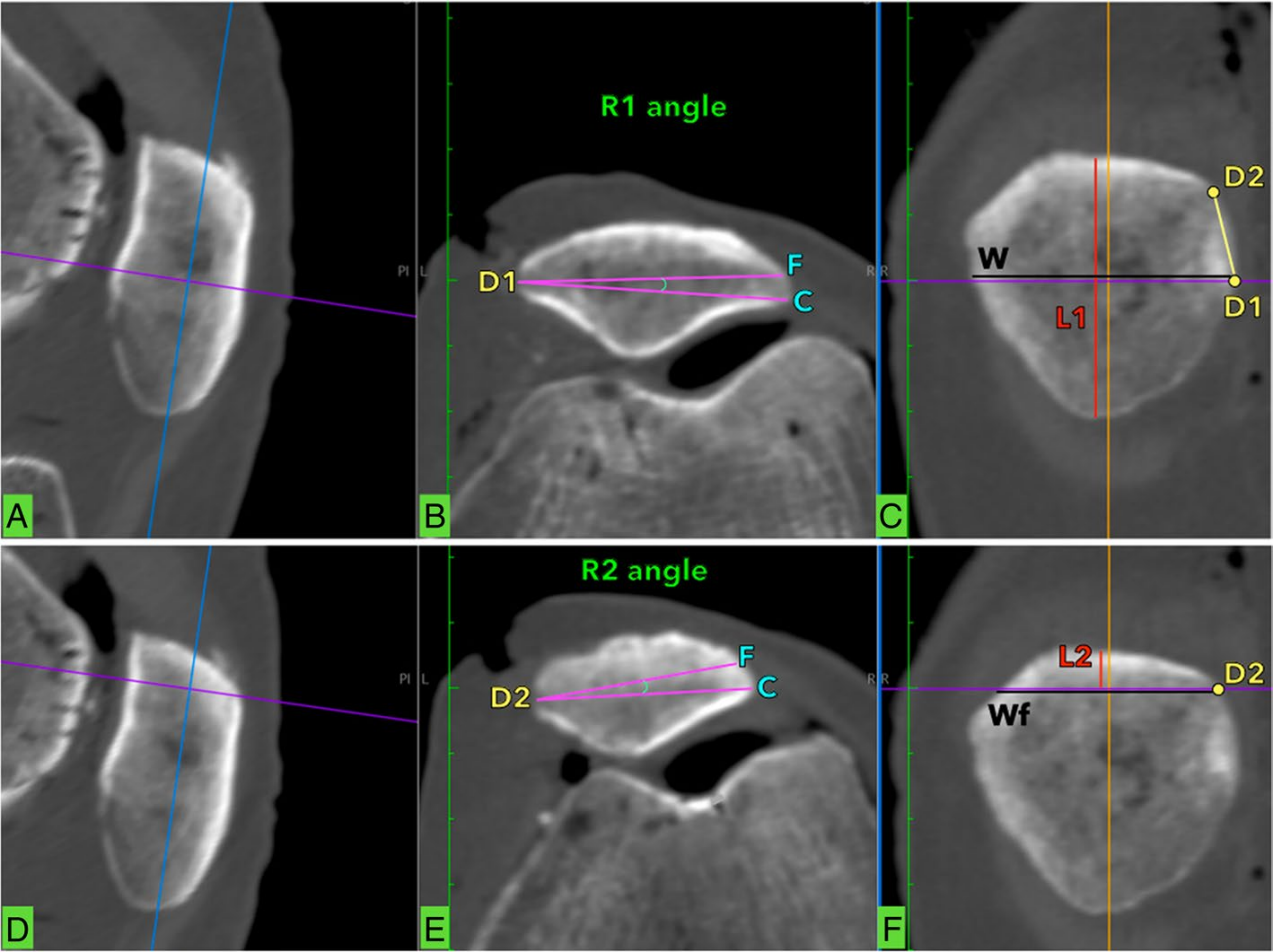
Preoperative planning. **a** Midsagittal section of the patella, blue and purple axis intersect at distal tunnel direction. **b** Midaxial section of the patella, F is the point of the rim of the anterior cortex at the lateral patella margin and C is the point of the rim of articular surface at the lateral patella margin. R1 is defined as the angle between F-D1-C. **c** Midcoronal section of the patella, L1 is the maximal patellar length. W is defined as patellar width (perpendicular to and at midpoint of L1). D1 is the distal tunnel entry point (where W touches the medial patellar margin) and D2 is the proximal tunnel entry point. **d** Midsagittal section of the patella, blue and purple axis intersect at proximal tunnel direction. **e** R2 is the angle between F-D2-C. **f** Wf is patella width at the point which a line parallel to W reaches the anterior cortex of proximal patella pole. D2 is the proximal tunnel entry point (where Wf touches medial patella margin). L2 iss the distance of proximal patella pole from Wf

**Fig. 2:**
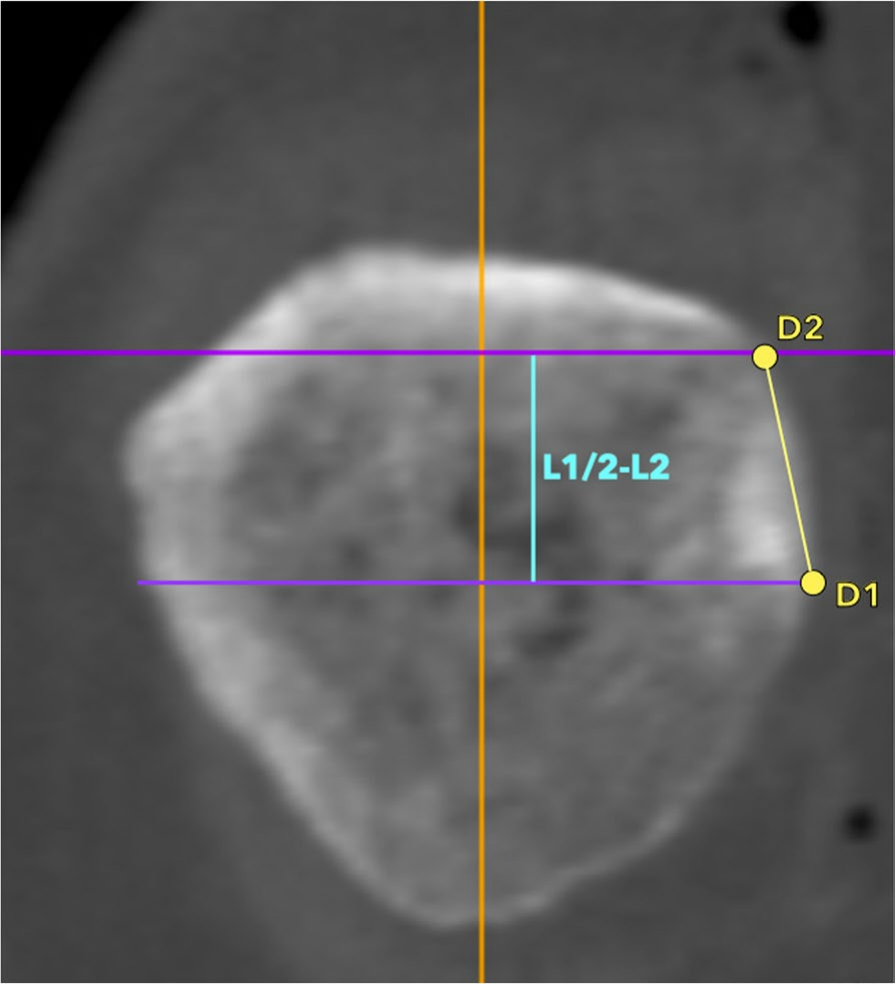
Preoperative planning (2). The maximum bone stock between tunnels is calculated as half of L1 minus the L2 distance (L1/2-L2). Consequently, D1-D2 distance is the maximum possible distance between the entry points of the two tunnels at the medial patella margin that the surgeon can perceive by palpation in real time surgical conditions

Midcoronal section definitions:

L1 was the maximal patellar length.

Considering a) the shape of the patella, b) the insertion of the quadriceps tendon and c) transverse parallel tunnel placement, L2 had to represent the minimum possible distance from the proximal pole of the patella for placement of the upper tunnel. This was defined as the distance between the proximal pole and to the superior border of the anterior cortex (Fig. 1d).

D1 was defined as the distal tunnel entry point (on the medial patellar surface). This was based at the patella midline or half of L1 distance (Fig. 1c).

D2 was defined as the entry point (on the medial patellar surface) for the proximal tunnel entry point. This was based at the point where a transverse line to the distal point of L2 touches medial patella margin (Fig. 1f).

The maximum bone stock between tunnels was calculated as half of L1 minus the L2 distance (L1/2-L2) (Fig. 2). Consequently, D1-D2 distance was the maximum possible distance between the entry points of the two tunnels at the medial patella margin that the surgeon can perceive by palpation in real time surgical conditions (Fig. 1c).

MidAxial section definitions:

F was the point of the rim of the anterior cortex at the lateral patella margin and C was the point of the rim of articular surface at the lateral patella margin. R1 was defined as the angle between F-D1-C (Fig. 1b) and R2 as the angle between F-D2-C (Fig. 1e). R angles represent safe angles at the entry point during tunnel drilling in MPFL reconstruction (without breaching the anterior cortex or articular cartilage).

### Technique

The technique with semi-patella tunnels was chosen because it avoids breaching the anterior cortex of the patella, utilizes blind transverse tunnels (not trans-patellar tunnels) minimizing the tunnel size, and provides aperture fixation with tendon-to-bone tunnel healing [32].

Gracilis tendon autograft was harvested through a vertical incision placed 2 cm medially to the pes anserinus. After the preparation of the gracilis tendon graft (approximately 20–21 cm), a running locking Krackow suture was placed up to approximately 2 cm from each free end with a Νo. 2 non-absorbable suture (Ethibond). With the knee flexed at 90°, a second longitudinal incision (2–3 cm) was performed on the anteromedial side of the patella and the medial aspect of the patella was exposed all the way to the bone surface by electrocautery, without penetrating the capsule. A guide pin of 2.0-mm diameter with an eyelet was transversely inserted from the midpoint of the medial edge of the patella (by palpations and lateral x-ray) to the lateral border, a) free hand (Group A) and b) with the help of an ACL tibia aiming device to avoid breaching either the articular surface or the anterior cortex (Group B). Intra-operatively, an anteroposterior x-ray is not helpful for guide wire positioning and measuring the distance between tunnels, because the patella is obscured by the distal femur. The direction guide pin was drilled in a transverse fashion, perpendicular to the longitudinal axis of the patella and parallel to the coronal patella plane. The appropriate placement of the guide pin was confirmed by fluoroscopy. Distal drilling was performed first. A second guide pin was placed at least 10 mm proximally and parallel to the first pin, as checked using a ruler and the two guide pins were over-drilled with a cannulated 4.5-mm drill bit 2-cm deep, to create two 2-cm transverse bone tunnels at the medial side of the patella. The appropriate placement of the second guide pin was also confirmed by fluoroscopy. Two suture loops were inserted into the tunnels, with the loop lying on the medial side.

The knee is then flexed to 30°, and the adductor tubercle was identified by palpation and under fluoroscopic guidance, a 2.4-mm guide pin with an eyelet is drilled at the Schöttle point. Afterwards, the guide pin was over-reamed with a 6-mm cannulated reamer to a depth of 30 mm. The prepared graft was passed through the patellar incision, so that the sutures of each free graft-end were passed through the suture-loops at the patella tunnels and then pulled out from medial to lateral. Both ends of the tendon graft were pulled into the 2 patella tunnels, and the graft sutures were tied together with tension for stable graft fixation at the lateral patella rim. The graft loop was pulled into the created femoral tunnel for 2 cm or more and was finally fixed with a 7-mm interference screw at 20–30° of knee flexion.

### Postoperative measurements

For the comparison of postoperative patella tunnels placement with preoperative planning, the following parameters were evaluated for all MPFL reconstructions (without grouping): 1) the transosseous bone bridge between the two sutures (BBS), 2) the transosseous bone bridge between tunnels (BBT), 3) whether tunnels were parallel to each other and 4) violation of the articular surface or the anterior cortex.

Sample size requirement was calculated to be *N* = 15 for each group, which corresponds to 0.8 power [21]. Student’s t-test and the correlation coefficient (r) were used for comparison between groups. Excel for Mac software was used to statistically compare experimental results. The level of significance was set at *p* < 0.05. Interobserver agreement for measurements was tested between two equally experienced orthopaedic surgeons. Prior to the actual agreement study, consensus was reached on the measurement protocol. Bias due to difference of equipment (e.g., different screen size and analysis) was eliminated by using the same radiologist workstation. Each observer was blinded to the other observer’s measurements for the interobserver agreement analysis. For the intraobserver analysis, one observer was blinded to his own prior measurements and there was an 8-week interval between his first and second measurements. Intraclass correlation coefficient (ICC) was used to determine both inter- and intraobserver agreement.

## Results

**Fig. 3:**
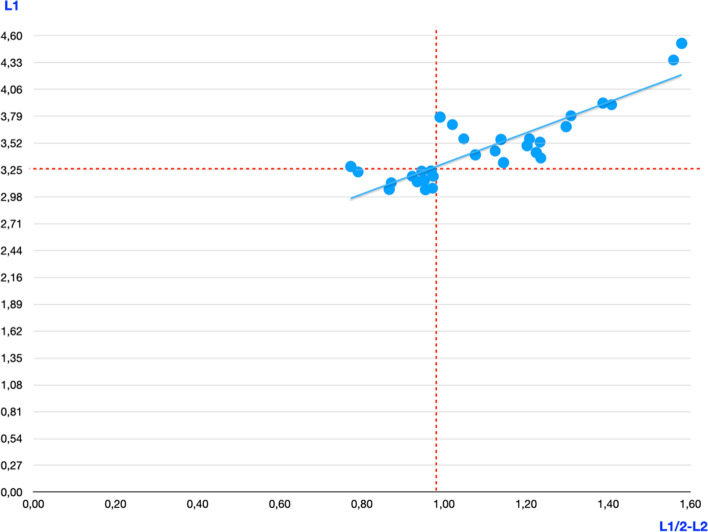
Graph, correlation between L1 and L1/2-L2. y axis is L1 and x axis is L1/2-L2. Perpendicular red dotted line represents the maximum bone bridge between tunnels equal to 1 cm and horizontal dotted line represents patella length equal to 3.25 cm. The blue line represents the correlation coefficient. Patellas shorter than 3.25 cm correspond to bone bridge shorter than the “desired” 1 cm length

**Fig. 4:**
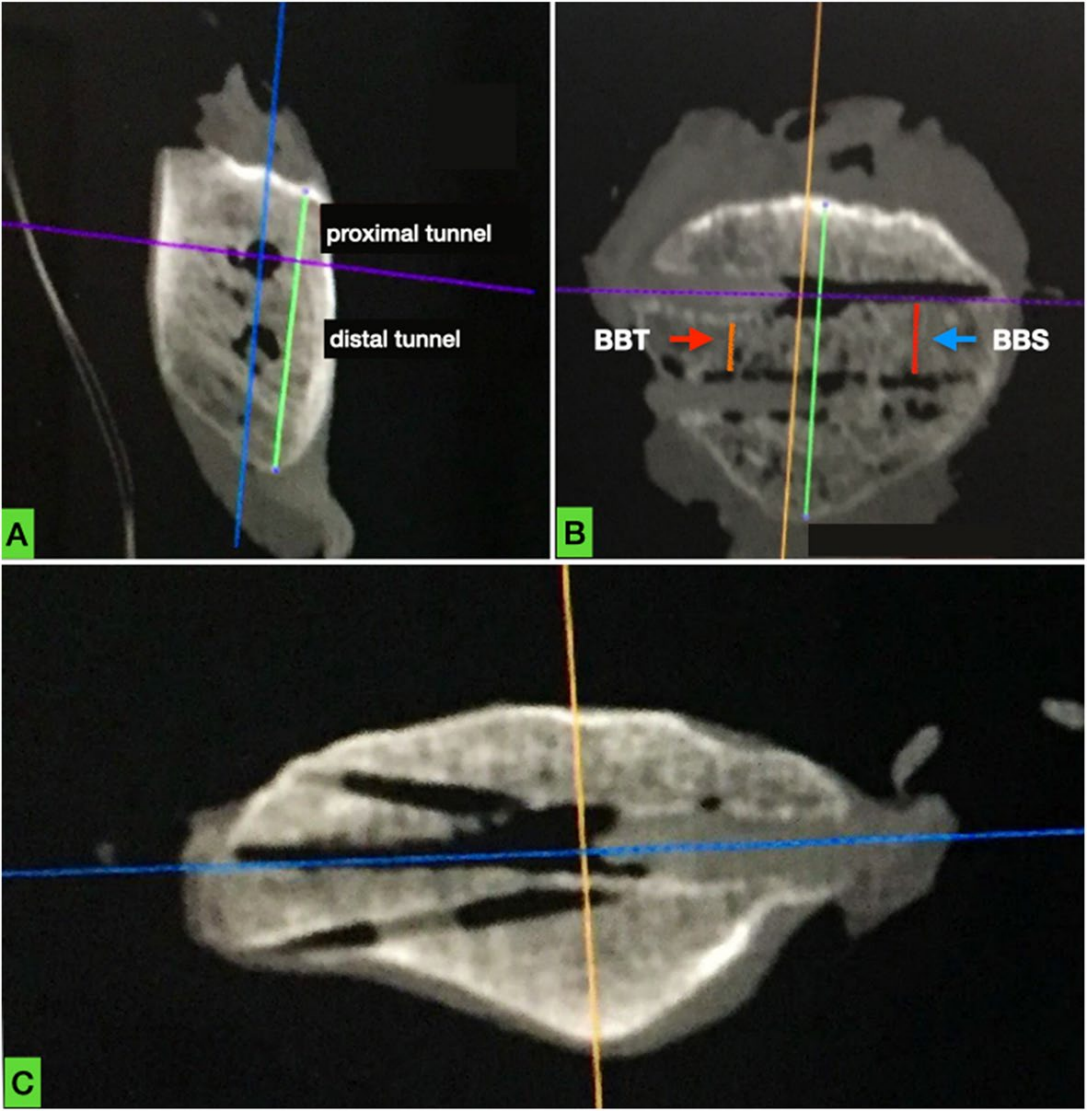
Sagittal, frontal and axial CT section of patella #7 postoperatively. Tunnel placement in **a** and **b** appears adequate. However, in **c**, multiple attempts (with breaching of the articular surface and the anterior cortex) made by the surgeon for proximal tunnel placement are revealed. Blue arrow in **b** points at the postoperative bone bridge (red line) between transosseous sutures (BBS). Red arrow in **b** points at the postoperative bone bridge (orange line) between tunnels (BBT)

**Fig. 5:**
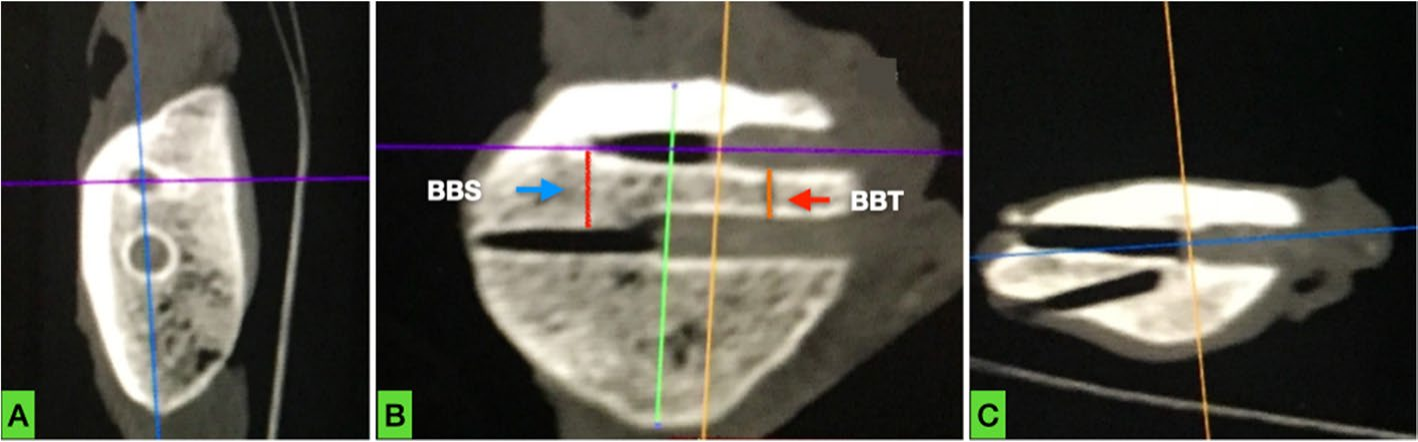
Sagittal, frontal and axial CT section of patella #10 postoperatively. Tunnel placement in **a** and **b** appears adequate. However, in **c**, two attempts made by the surgeon for proximal tunnel placement are revealed. The surgeon was probably unable to create a correct tunnel between previous attempted drillings. Blue arrow in **b** points at the postoperative bone bridge (red line) between transosseous sutures (BBS). Red arrow in **b** points at the postoperative bone bridge (orange line) between tunnels (BBT)

Preoperatively, mean patella length (L1) was 3.45 cm (range 3.05–4.52, SD 0.39). Mean L2 was 0.62 cm (range 0.49–0.89, SD 0.12). The mean maximum possible bone stock between tunnels (L1/2-L2) was 1.1 cm (range 0.77–1.58, SD 0.21) and D1-D2 distance was 1.19 cm (range 0.95–1.67, SD 0.24) (Table 1). L1/2-L2 was significantly shorter than D1-D2 (*p* = 0.035). The correlation coefficient for L1 and D1-D2 was *r* = 0.78 and for L1 and L1/2-L1 it was *r* = 0.82. Consequently, bone bridge between tunnels in patellas shorter than 3.25 cm, was less than 1 cm (Fig. 3). R1 was 6.05^0^ (range 4.78–7.44, SD 0.92), R2 was 6.64^0^ (range 4.57–9.03, SD 1.03), and their difference reached statistical significance (*p* = 0.03). Postoperatively, in group A, in 4 out of 15 patellas, multiple attempts were made during tunnel drilling in order to avoid anterior cortex or cartilage breaching (Figs. 4 and 5). In group B, all tunnels were correctly drilled with the first attempt (within angle between points F and C at the lateral surface of the patella).

**Table 1:** CT Measurements (preoperative and postoperative)

	**R1**	**R2**	**L1 (cm)**	**L2 (cm)**	**D1-D2 (cm)**	**L1/2-L2 preop (cm)**	**Bone bridge between sutures (BBS)**	**Bone bridge between tunnels (BBT)**
Mean	6.048	6.64	3.44	623	1.19	1.09946	0.93	0.4706
Range	4.78–7.44	4.57–9,03	3.05–4.52	0.49–0.89	0.95–1.67	0.87–1.58	0.7–0.21	0.3–0.75
SD	0.92	1.03	0.39	0.12	0.24	0.2	0.14	0.15

Mean BBS was significantly shorter postoperatively (0.93 cm, range 0.7–1.21, *p* < 0.01). Mean BBT was 0.47 cm (range 0.3–0.75) (Table 1). All tunnels in both groups were drilled in a parallel fashion.

For intraobserver agreement, ICC was 0.992 with 95% CI between 0.990 and 0.994 and 0.990 for interobserver agreement, with 95% CI between 0.988 and 0.993 (excellent agreement beyond chance [5]).

## Discussion

This study introduces safe angles (R1 and R2) for tunnel drilling in MPFL reconstruction and highlights their clinical importance. R1 and R2 angles have never before been described, correlated or calculated in published literature, and their small range has important clinical implications. Furthermore, the ACL tibia device appears to aid in safe drilling of transverse transpatellar tunnels during MPFL reconstruction. Finally, from preoperative-postoperative comparisons, we found that the bone bridge between tunnels, in order to create an anatomic, double-bundle MPFL reconstruction, appears to be directly related to the patella length (Fig. 3) and could create technical difficulties in small-sized patellae.

R angles have a relative small range, which translates into a narrow window for safe manipulations during tunnel drilling in order to avoid violating the anterior cortex or articular surface. This has clinical applications for any surgical technique utilizing patella bone tunnels, such as distal pole patella fracture fixation [1]. We found R1 angle to be significantly smaller than R2. As a result, the surgeon should proceed more cautiously during drilling of the distal tunnel. Breaching of the anterior patella cortex has been incriminated for complete patella fracture after MPFL reconstruction [24–26, 36, 40]. In this study, thanks to the ACL tibia device, no breaching or multiple attempts at drilling were observed during the placement of 30 tunnels in 15 patellas of group B, and this finding emphasizes the usefulness of the aiming device for more accurate tunnel drilling. Considering the small size and the special shape of the patella, it is very important to drill two parallel transverse semi-patellar tunnels with the first attempt, so that the bony integrity of the patella is not compromised. Therefore, the ACL tibia device appears to create safer conditions for tunnel drilling during MPFL reconstruction. An alternative but time-consuming option would be to perform an intraoperative Merchant view x-ray during tunnel drilling, not to mention increasing radiation exposure.

In our study, the mean patella length (L1) was 3.45 cm (ranging from 3.05–4.52 cm), which corresponds to smaller patella size compared to the values published in the literature [8, 9, 18, 37]. Previous reports on the double bundle MPFL reconstruction techniques describe tunnel placement in the upper half of the patella and distance between tunnels to be at least 1 cm [16, 27, 29–34]. Preoperatively, when mapping ideal patella tunnel placement with the use of preoperative CT scan, we found 12 out of 30 patellae to have maximum bone bridge (L1/2-L2) between tunnels under 1 cm, and their length (L1) was less than 3.25 cm. Collectively, preoperative mean maximum possible bone bridge with ideal tunnel placement was calculated to be 1.1 cm. Postoperatively, mean BBS was 0.93 cm, significantly shorter than preoperative planning, possibly owing to the use of D1-D2 distance for tunnel placement instead of L1/2-L1 by the surgeon. As previously mentioned, intra-operative anteroposterior x-ray is not helpful because the patella is obscured by the distal femur. BBS under 1 cm could lead to bone bridge collapse and consequently to MPFL reconstruction failure [29]. A bone bridge collapse between tunnels could transform double-bundle technique to single-bundle. No such collapse was observed in this study, but we are unsure of its integrity and sufficiency after exertion of in vivo forces. To sum up, the double-bundle, transpatellar tunnels, free-implant technique should be used cautiously in small patellae (caucasian outliers or pediatric or Asian population). Other solutions include double-bundle fixation technique with anchors or non-anatomic patella fixation with a single tunnel [22] or the use of quadriceps graft [4].

This study is not without limitations. Clinical implications and conclusions should be drawn cautiously, since this is a cadaveric study, with a relative small sample size. Another important limitation is the old age of cadavers used in the study. Despite methodological limitations, this is an original, cadaveric study, offering new insight on the patella surgical and radiological anatomy, that could affect current surgical practice.

## Conclusion

Small-size patellae correlate with short maximum bone bridge between tunnels, which makes anatomic, double-bundle, hardware-free patella fixation, with two semi-patellar tunnels MPFL reconstruction challenging. Furthermore, R angles create a narrow window to avoid intraoperative breaching, rendering the use of the ACL tibia device an extremely useful instrument.
